# A Predictive Coding Perspective on Beta Oscillations during Sentence-Level Language Comprehension

**DOI:** 10.3389/fnhum.2016.00085

**Published:** 2016-03-03

**Authors:** Ashley G. Lewis, Jan-Mathijs Schoffelen, Herbert Schriefers, Marcel Bastiaansen

**Affiliations:** ^1^Haskins Laboratories, New HavenCT, USA; ^2^Neurobiology of Language Department, Max Planck Institute for PsycholinguisticsNijmegen, Netherlands; ^3^Center for Cognitive Neuroimaging, Donders Institute for Brain, Cognition and Behaviour, Radboud UniversityNijmegen, Netherlands; ^4^Donders Center for Cognition, Donders Institute for Brain, Cognition and Behaviour, Radboud UniversityNijmegen, Netherlands; ^5^Academy for Leisure, NHTV Breda University of Applied SciencesBreda, Netherlands

**Keywords:** language comprehension, neural oscillations, beta, predictive coding, EEG, MEG

## Abstract

Oscillatory neural dynamics have been steadily receiving more attention as a robust and temporally precise signature of network activity related to language processing. We have recently proposed that oscillatory dynamics in the beta and gamma frequency ranges measured during sentence-level comprehension might be best explained from a predictive coding perspective. Under our proposal we related beta oscillations to both the maintenance/change of the neural network configuration responsible for the construction and representation of sentence-level meaning, and to top–down predictions about upcoming linguistic input based on that sentence-level meaning. Here we zoom in on these particular aspects of our proposal, and discuss both old and new supporting evidence. Finally, we present some preliminary magnetoencephalography data from an experiment comparing Dutch subject- and object-relative clauses that was specifically designed to test our predictive coding framework. Initial results support the first of the two suggested roles for beta oscillations in sentence-level language comprehension.

## Introduction

Language comprehension requires the fast and efficient integration of information represented at a multitude of different levels and timescales (Jackendoff, 2007). This means that numerous different and often spatially distant brain regions have to interact quickly and dynamically in order to achieve even the most basic linguistic processing. It is therefore not surprising that oscillatory neural dynamics have been steadily receiving more attention as a robust and temporally precise signature of network activity related to language processing (e.g., Weiss and Mueller, 2012; Friederici and Singer, 2015; Lewis et al., 2015). We have recently suggested a role for beta and gamma oscillations in supporting sentence-level language comprehension (Lewis and Bastiaansen, 2015). In this article we zoom in on the role that beta oscillations play in our proposal, reviewing the available evidence old and new, and presenting some preliminary findings from an experiment designed to directly test one of our hypotheses.

## The Proposal In A Nutshell

Our proposal (for a detailed outline see Lewis and Bastiaansen, 2015) suggests that oscillatory neural activity in the beta frequency range (13–30 Hz) during sentence-level language comprehension reflects both the active maintenance/change of the underlying neurocognitive network (NCN; Bressler and Richter, 2014) responsible for the representation and construction of the current sentence-level meaning, and the top–down propagation of predictions from higher to lower levels of the cortical hierarchy based on that sentence-level meaning. When the language comprehension system actively maintains the current mode of processing, beta activity within the associated NCN should increase, while a change in the current mode of processing should result in a decrease in beta activity within that NCN (Engel and Fries, 2010; Lewis and Bastiaansen, 2015; Lewis et al., 2015). Similarly, for predictions about upcoming linguistic information with high levels of certainty, beta activity in the NCN should increase in a direction-specific manner (from higher to lower levels of the cortical processing hierarchy; Bastos et al., 2012; Friston et al., 2014). If there are cues in the linguistic input indicating that the current mode of processing is expected to change, the language comprehension system should place less emphasis on top–down predictions, which in turn should result in a decrease in top–down beta activity (Bastos et al., 2012; Friston et al., 2014; Lewis and Bastiaansen, 2015). Such a role for beta in top–down signaling of predictions based on a generative model within a predictive coding framework has been proposed outside of the domain of language comprehension (Bastos et al., 2012; Friston et al., 2014), and we simply apply these ideas to sentence-level comprehension. It may turn out that certain aspects of these two suggested roles for beta activity are complementary, while others are incompatible. The evidence reported here (see If the Evidence Fits … and (If the Evidence Fits…) Test the Hypothesis) does not allow us to differentiate between them. We would like to make it explicit that we are not arguing for a relationship between beta activity and measure of word surprisal (cf. Levy, 2008), although it is entirely possible that such a relationship may exist.

Before moving on to examine evidence from previous literature, we think it is important to specify exactly what we mean by top-down predictions. In our opinion a clearer distinction has to be made between predictions at the cognitive level and predictions at the neural level. At the cognitive level, and for sentence-level language comprehension in particular, we consider prediction to refer to the activation of specific lexical information stored in long-term memory prior to the appearance of that information in the linguistic input stream (e.g., DeLong et al., 2005; Van Berkum et al., 2005; Szewczyk and Schriefers, 2013; see also Huettig, 2015 for discussion). On the other hand, within the domain of predictive coding implementations of hierarchical Bayesian inference in the brain, predictions are nothing more (but nothing less) than the neural activity at representational units at a ‘higher’ hierarchical level, that is propagated down to the error units at a hierarchically ‘lower’ level (Friston, 2005). This neural activity may sometimes directly correspond to prediction at the cognitive level, but most often it will not, because cognitive predictions, and neural predictions generally operate on different timescales. Neural predictions are updated in an ongoing fashion based on numerous factors, including prediction errors sent up the cortical processing hierarchy. Predictions at the cognitive level likely involve evidence accumulation over time until some critical threshold is reached, after which lexical (or more generally long-term memory) pre-activation occurs. This lexical pre-activation may in turn serve as a neural prediction signal that influences activity at lower levels of the cortical hierarchy. Conflating prediction at the cognitive and at the neural level can often lead to confusion in discussions of predictive processing. Our proposal relating beta to top-down prediction refers to predictions at the neural level, but allows for the possibility that predictive processing at the cognitive level may drive these neural prediction signals.

## If The Evidence Fits …

Next we turn our attention to the evidence supporting our proposed role for beta oscillations during sentence-level language comprehension. We start by briefly summarizing the evidence we have already reviewed elsewhere (Lewis and Bastiaansen, 2015; Lewis et al., 2015), and then move on to discuss one new piece of evidence.

There are by now a number of studies reporting that beta power is sensitive to both syntactic violations (Davidson and Indefrey, 2007; Bastiaansen et al., 2010; Pérez et al., 2012; Kielar et al., 2014) and semantic incongruities (Luo et al., 2010; Wang et al., 2012; Kielar et al., 2014). In all of these studies, beta power was higher following some target word for syntactically and semantically acceptable sentences compared to target words that resulted in a syntactic violation or a semantic incongruity. Similarly, Luo et al. (2010) showed that beta power was higher for rhythmically normal compared to abnormal target nouns in Chinese verb-noun pairs. In addition to grammatical violations, Pérez et al. (2012) showed that beta power following a target word was lower for the case of Spanish ‘Unagreement’ (where the sentence remains grammatical despite a mismatch between the grammatical person feature marking on the subject and that on the verb of a sentence) compared to grammatically legal target words. These studies all have in common that there is some cue in the linguistic input (e.g., syntactic violation, semantic incongruity, etc.) that indicates to the language comprehension system that the current representation of the sentence-level meaning is not correct and needs to be changed. We suggest that the result is a change in the NCN responsible for that representation, and that this leads to a decrease in beta power in that NCN (or in one or multiple nodes of that network). It may also result in the system assigning less value to top–down predictions as that information has proven unreliable, which would also result in a decrease in beta activity.

Another group of studies has shown that beta activity is higher when sentences are more syntactically demanding, but still grammatical (Weiss et al., 2005; Bastiaansen and Hagoort, 2006; Meyer et al., 2013). Bastiaansen and Hagoort (2006) reported that beta power was higher for syntactically more demanding center-embedded compared to right-branching relative clauses. Meyer et al. (2013) showed that beta power was higher for long- compared to short-distance subject-verb agreement dependencies at the point in the sentences where the dependency could be resolved. Weiss et al. (2005) found higher beta coherence between frontal and posterior electrode sites for syntactically more complex object-relative (OR) compared to subject-relative (SR) clauses. We suggest that in all these cases the increased beta activity reflects the active maintenance of the current NCN configuration responsible for the construction and representation of the current sentence-level meaning. It may also indicate a greater reliance on top-down predictions based on that sentence-level meaning (i.e., the increased activity may be related to greater weighting of the top-down signal based on the current generative model), in order to actively try to integrate the new linguistic input into the current sentence-level meaning representation.

Bastiaansen et al. (2010) showed that beta power increased linearly over the course of syntactically legal sentences, but returned to baseline levels at the point of a syntactic violation within sentences. They also showed that lists of the same words contained in the sentences in random order (no syntactic structure) did not exhibit any increase in beta power over the course of presentation of the lists (see also Bastiaansen and Hagoort, 2015). We suggest that the gradual buildup of beta power over the course of sentences might be related to the gradually increasing activation of a NCN responsible for the construction and representation of the sentence-level meaning, and that this network becomes disengaged upon reaching a syntactic violation resulting in the decrease in beta power at that point. For random word lists no sentence-level meaning can be constructed, and thus beta power does not increase over the course of their presentation.

Finally, Magyari et al. (2014) presented participants with natural speech, where the ends of speaking turns were either highly predictable or unpredictable, and asked them to press a button when they thought a speaker’s turn was about to end. They showed a decrease in beta power just before a button press in the highly predictable condition and an increase in beta power in the unpredictable condition. We suggest that the decrease in beta power in the predictable condition occurs because the language comprehension system anticipates that the current processing mode will have to change (from comprehending the sentence to giving a meta-linguistic judgment by making a button press). In the unpredictable condition the language comprehension system does not predict that the processing mode will change, and instead the current sentence-level meaning representation is actively maintained, resulting in the increased beta power in that condition.

There is one new beta finding that was not included in our previous reviews. Kielar et al. (2015) have followed up on their EEG study investigating syntactic violations and semantic anomalies compared to control sentences (Kielar et al., 2014) by adding conditions with auditory stimulus presentation (the original used only visual presentation), and by using a beamforming approach [in this case applied to magnetoencephalography (MEG) data] to obtain more precise information about the spatial extent of their effects. They replicate the finding of higher beta (and alpha; see Kielar et al., 2015 for details) power for control sentences compared to both syntactic violations and semantic anomalies, this time for both the visual, and auditory input modalities. Furthermore, their source localization results (albeit computed for the broadband data in the alpha and beta frequency ranges combined; 8–30 Hz) implicated what are arguably the main nodes of the core language network (e.g., Hagoort, 2005, 2013; Hickok and Poeppel, 2007), namely left inferior frontal regions, left superior temporal cortex, and left angular and supramarginal gyri. Our suggestion that when a syntactic violation or semantic incongruity is encountered, decreased beta power reflects a change in the NCN responsible for the representation and construction of a sentence-level meaning holds here as well. However, this study makes an important next step by more precisely mapping out the cortical regions involved. In our opinion, the use of source reconstruction techniques with electrophysiological data is important in future language comprehension studies in order to gain more fine-grained insights into the spatial distribution of the cortical networks whose temporal dynamics are being investigated. At this stage we can only speculate that the critical cortical nodes comprising the NCN that supports sentence-level language comprehension include the core language regions mentioned above. Depending on the context in which language comprehension takes place, this network may interact with other cortical networks like the attention network (e.g., in case the listener/reader finds themselves in a particularly distracting environment) or the theory of mind network (e.g., when interacting with a conversation partner). Working out these details is one important avenue for future investigation.

## (If The Evidence Fits…) Test The Hypothesis

So far, all evidence presented in favor of our hypothesis is based on a re-interpretation of the results of studies that were not specifically designed to test the hypothesis that beta power is related to the maintenance/change of the NCN responsible for representing a sentence-level meaning. Now we present some preliminary data from a MEG experiment that was designed to test this hypothesis. Participants read Dutch SR and OR clause sentences, where the input was identical up to an auxiliary verb presented at the end of the relative clause, disambiguating between the two relative clause types (see **Table 1** for example stimuli). The auxiliary could agree in grammatical number with either the referent in the matrix clause (SR) or with the referent in the relative clause (OR). There was no information in the linguistic input prior to the auxiliary that provided any indication about whether the sentence should be read as a subject- or an object-relative clause (the past participle did not bias the reader to have a preference for either of the possible referents). In all cases both referents were animate and the verb in the relative clause was not semantically biased toward having either of the two referents as a grammatical subject. Dutch readers show a clear preference for the SR reading of these sentences, which appears more frequently in Dutch corpora (Mak et al., 2002). This means that Dutch readers typically parse the sentence as a SR clause, and when encountering linguistic input indicating that it should instead be read as an OR clause they experience a disruption in processing, resulting in longer reading times (Mak et al., 2002, 2006, 2008). We hypothesized that this ‘unexpectedness’ of the OR reading should result in a decrease in beta power at the disambiguating auxiliary at the end of the relative clause compared to the SR condition (as an aside, one may wonder why this is not also predicted in the case of the center-embedded compared to right-branching relative clause sentences reported in Section “If the Evidence Fits …,” but in that case the center-embedded relative clause sentences were not unexpected). This is hypothesized because although the sentence is grammatical, there is a cue in the linguistic input (a mismatch between the grammatical number feature on the verb and the grammatical number feature on the expected referent), which indicates that the current representation of the sentence-level meaning (and therefore the underlying NCN) needs to change.

**Table 1:** Example materials used in preliminary experimental findings reported and their direct English translation (in italics).

Condition	Example materials
SR	Ongerust kijkt de hardloper, die de wandelaars in het park gegroet heeft, naar de regenwolken in de lucht.
	*Anxiously looks the runner, that the walkers in the park greeted has, at the rainclouds in the sky.*
OR	Ongerust kijkt de hardloper, die de wandelaars in het park gegroet hebben, naar de regenwolken in de lucht.
	*Anxiously looks the runner, that the walkers in the park greeted have, at the rainclouds in the sky.*


*SR, subject-relative clause condition; OR, object-relative clause condition; auxiliary verb and referent that agrees with it in grammatical number are underlined.*

**FIGURE 1:**
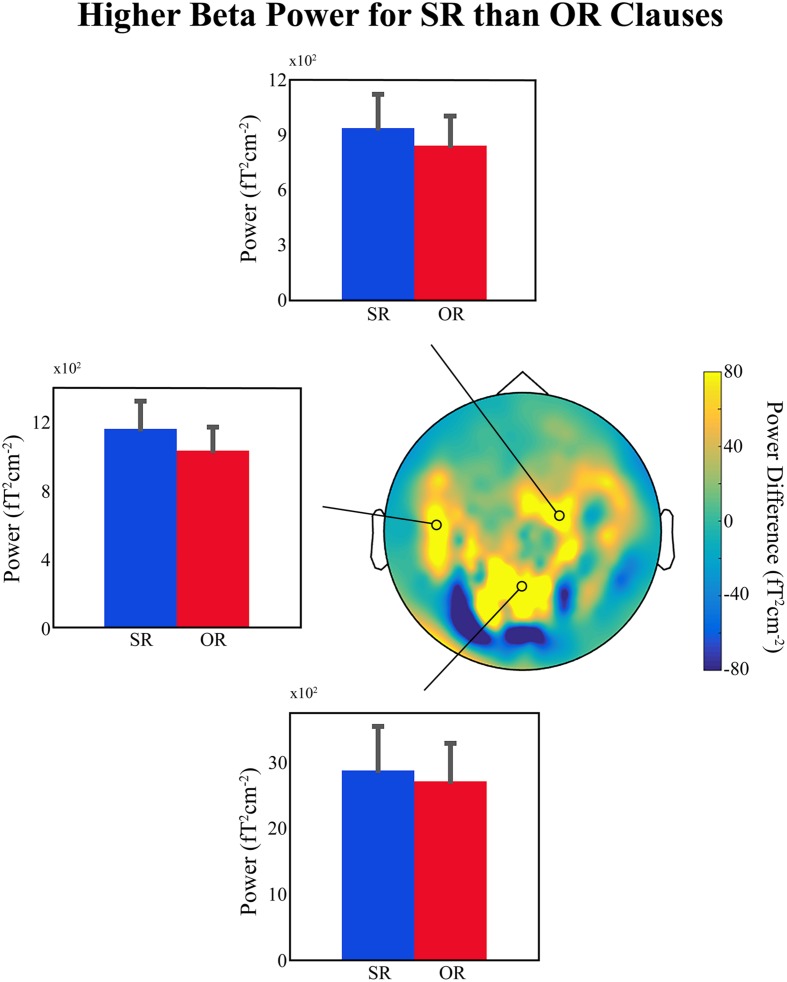
**Preliminary MEG findings.** (Top, Middle Left, and Bottom) Bar plots showing the average beta power (12–16 Hz) for the subject-relative (SR) and object-relative (OR) conditions in a region between 750 and 1050 ms relative to the onset of the disambiguating auxiliary verb, and at three representative sensors. Error bars indicate standard error of the mean. (Middle Right) Topographical distribution of the difference in power (SR – OR) for the selected beta time-frequency range. Position of selected representative sensors indicated by black circles. Data presented were high-pass filtered above 0.1 Hz and artifacts related to power-line interference, superconducting quantum interference device (SQUID) jumps, muscle activity, eye-movements, eye-blinks, and cardiac activity were removed. The planar gradient representation of the data for each participant (25 in total – written ethical approval was obtained) was computed and a time-frequency decomposition was carried out using a series of Slepian tapers (Mitra and Pesaran, 1999), and a sliding-window approach in time steps of 20 ms and frequency steps of 2 Hz. Time windows of 500 ms and frequency smoothing of 4 Hz were employed. Data were then averaged over the time and frequency ranges of interest (see above) separately for each condition, and grand-averages across all participants were computed for comparison.

**Figure 1** shows bar plots of the average power at selected MEG sensors in the beta frequency range (12–16 Hz), between 750 and 1050 ms relative to the onset of the disambiguating auxiliary, for the two conditions. There is a small but clear difference in lower-beta power over left temporal and right frontal regions (also present but much less clear over centro-parietal sensors; top, left middle, and bottom panels, respectively, in **Figure 1**), with higher beta power for SR compared to OR clauses, exactly as predicted. This provides further support for the idea that a decrease in beta power is related to the ‘unexpectedness’ of the incoming linguistic input, regardless of whether or not the sentence becomes ungrammatical or semantically anomalous. Added to the findings reviewed in Section “If the Evidence Fits …,” the available evidence suggests that upon encountering unexpected linguistic input the language comprehension system prepares for a change in the current mode of processing, and a change in the NCN responsible for representing the current sentence-level meaning. This change is reflected in a decrease in beta power in the underlying NCN (or in certain nodes of that NCN). We would like to emphasize that since this is only preliminary data it should only be considered tentative support for our hypothesis.

The beta power decrease may also reflect diminished ‘confidence’ in top-down predictions by the language comprehension system after encountering unexpected linguistic input. Our experiment does not directly address hypotheses about beta carrying top–down predictions, but it is possible that the local modulations of beta power do reflect such predictions. In order to directly test the hypothesis about top–down information in a predictive coding framework one first needs to define the different hierarchical levels involved at the cognitive level (e.g., a unification component sending predictions down the hierarchy to a memory component; cf. Hagoort, 2005, 2013) and the cortical regions responsible for instantiating those cognitive components (e.g., left inferior frontal cortex, and left temporal cortex). Then a directional measure of oscillatory activity (e.g., Granger causality, dynamic causal modeling, or transfer entropy; Friston et al., 2013; Park et al., 2015) can be used to directly test whether or not beta activity is predominant from higher to lower levels of the cortical hierarchy (e.g., from left inferior frontal cortex to left temporal cortex).

## Conclusion

In this article we have zoomed in on our proposed role for oscillatory activity in the beta frequency range in both the maintenance/change of the NCN underlying the construction and representation of a sentence-level meaning, and the propagation of top-down predictions to lower levels of the cortical processing hierarchy based on that sentence-level meaning. We reviewed old and new evidence supporting our proposed roles for beta, and presented some preliminary findings from an experiment designed to directly test one of our hypotheses. The results make a compelling case for beta as an index of maintenance/change of the current NCN underlying sentence-level meaning representation and construction. It will be important for future research to directly test the proposed role of beta in top–down predictions, to further specify which cortical nodes are incorporated into the NCN in different linguistic contexts, and to investigate the extent of overlap between the two potential roles for beta (maintenance and top–down predictions). Performing analyses at the level of cortical sources rather than at the sensor/electrode level will be an important part of this endeavor.

## Author Contributions

AL and MB conceived the structure of the article. AL, JS, HS, and MB wrote the manuscript. For the preliminary data presented, AL, JS, HS, and MB designed the experiment, AL collected the data, AL and JS analyzed the data.

## Conflict of Interest Statement

The authors declare that the research was conducted in the absence of any commercial or financial relationships that could be construed as a potential conflict of interest.

## References

[B1] Bastiaansen M. C. M. Hagoort P. (2006). Oscillatory neuronal dynamics during language comprehension. *Prog. Brain Res.* 159 179–196. 10.1016/S0079-6123(06)59012-0 17071231

[B2] Bastiaansen M. C. M. Hagoort P. (2015). Frequency-based segregation of syntactic and semantic unification during online sentence level language comprehension. *J. Cogn. Neurosci.* 27 2095–2107. 10.1162/jocn_a_00829 26042498

[B3] Bastiaansen M. C. M. Magyari L. Hagoort P. (2010). Syntactic unification operations are reflected in oscillatory dynamics during on-line sentence comprehension. *J. Cogn. Neurosci.* 22 1333–1347. 10.1162/jocn.2009.21283 19580386

[B4] Bastos A. M. Usrey W. M. Adams R. A. Mangun G. R. Fries P. Friston K. J. (2012). Canonical microcircuits for predictive coding. *Neuron* 76 695–711. 10.1016/j.neuron.2012.10.038 23177956 PMC3777738

[B5] Bressler S. L. Richter C. G. (2014). Interareal oscillatory synchronization in top-down neocortical processing. *Curr. Opin. Neurobiol.* 31C, 62–66. 10.1016/j.conb.2014.08.010 25217807

[B6] Davidson D. J. Indefrey P. (2007). An inverse relation between event-related and time-frequency violation responses in sentence processing. *Brain Res.* 1158 81–92. 10.1016/j.brainres.2007.04.082 17560965

[B7] DeLong K. A. Urbach T. P. Kutas M. (2005). Probabilistic word pre-activation during language comprehension inferred from electrical brain activity. *Nat. Neurosci.* 8 1117–1121. 10.1038/nn1504 16007080

[B8] Engel A. K. Fries P. (2010). Beta-band oscillations–signalling the status quo? *Curr. Opin. Neurobiol.* 20 156–165. 10.1016/j.conb.2010.02.015 20359884

[B9] Friederici A. D. Singer W. (2015). Grounding language processing on basic neurophysiological principles. *Trends Cogn. Sci. (Regul. Ed.)* 19 329–338. 10.1016/j.tics.2015.03.012 25890885

[B10] Friston K. (2005). A theory of cortical responses. *Philos. Trans. R. Soc. Lond. B Biol. Sci.* 360 815–836. 10.1098/rstb.2005.1622 15937014 PMC1569488

[B11] Friston K. Moran R. Seth A. K. (2013). Analysing connectivity with Granger causality and dynamic causal modelling. *Curr. Opin. Neurobiol.* 23 172–178. 10.1016/j.conb.2012.11.010 23265964 PMC3925802

[B12] Friston K. J. Bastos A. M. Pinotsis D. Litvak V. (2014). LFP and oscillations-what do they tell us? *Curr. Opin. Neurobiol.* 31C, 1–6. 10.1016/j.conb.2014.05.004 25079053 PMC4376394

[B13] Hagoort P. (2005). On Broca, brain, and binding: a new framework. *Trends Cogn. Sci. (Regul. Ed.)* 9 416–423. 10.1016/j.tics.2005.07.004 16054419

[B14] Hagoort P. (2013). MUC (Memory, Unification, Control) and beyond. *Front. Psychol.* 4:416 10.3389/fpsyg.2013.00416 PMC3709422 23874313

[B15] Hickok G. Poeppel D. (2007). The cortical organization of speech processing. *Nat. Rev. Neurosci.* 8 393–402. 10.1038/nrn2113 17431404

[B16] Huettig F. (2015). Four central questions about prediction in language processing. *Brain Res.* 626 118–135. 10.1016/j.brainres.2015.02.014 25708148

[B17] Jackendoff R. (2007). A parallel architecture perspective on language processing. *Brain Res.* 1146 2–22. 10.1016/j.brainres.2006.08.111 17045978

[B18] Kielar A. Meltzer J. Moreno S. Alain C. Bialystok E. (2014). Oscillatory responses to semantic and syntactic violations. *J. Cogn. Neurosci.* 26 2840–2862. 10.1162/jocn_a_00670 24893735

[B19] Kielar A. Panamsky L. Links K. A. Meltzer J. A. (2015). Localization of electrophysiological responses to semantic and syntactic anomalies in language comprehension with MEG. *Neuroimage* 105 507–524. 10.1016/j.neuroimage.2014.11.016 25463470

[B20] Levy R. (2008). Expectation-based syntactic comprehension. *Cognition* 106 1126–1177. 10.1016/j.cognition.2007.05.006 17662975

[B21] Lewis A. G. Bastiaansen M. C. M. (2015). A predictive coding framework for rapid neural dynamics during sentence-level language comprehension. *Cortex* 68 155–168. 10.1016/j.cortex.2015.02.014 25840879

[B22] Lewis A. G. Wang L. Bastiaansen M. C. M. (2015). Fast oscillatory dynamics during language comprehension: unification versus maintenance and prediction? *Brain Lang.* 148 51–63. 10.1016/j.bandl.2015.01.003 25666170

[B23] Luo Y. Zhang Y. Feng X. Zhou X. (2010). Electroencephalogram oscillations differentiate semantic and prosodic processes during sentence reading. *Neuroscience* 169 654–664. 10.1016/j.neuroscience.2010.05.032 20580785

[B24] Magyari L. Bastiaansen M. C. M. de Ruiter J. P. Levinson S. C. (2014). Early anticipation lies behind the speed of response in conversation. *J. Cogn. Neurosci.* 26 2530–2539. 10.1162/jocn_a_00673 24893743

[B25] Mak W. Vonk W. Schriefers H. (2006). Animacy in processing relative clauses: the hikers that rocks crush. *J. Mem. Lang.* 54 466–490. 10.1016/j.jml.2006.01.001

[B26] Mak W. M. Vonk W. Schriefers H. (2002). The influence of animacy on relative clause processing. *J. Mem. Lang.* 47 50–68. 10.1006/jmla.2001.2837

[B27] Mak W. M. Vonk W. Schriefers H. (2008). Discourse structure and relative clause processing. *Mem. Cogn.* 36 170–181. 10.3758/MC.36.1.170 18323073

[B28] Meyer L. Obleser J. Friederici A. D. (2013). Left parietal alpha enhancement during working memory-intensive sentence processing. *Cortex* 49 711–721. 10.1016/j.cortex.2012.03.006 22513340

[B29] Mitra P. P. Pesaran B. (1999). Analysis of dynamic brain imaging data. *Biophys. J.* 76 691–708. 10.1016/S0006-3495(99)77236-X 9929474 PMC1300074

[B30] Park H. Ince R. A. A. Schyns P. G. Thut G. Gross J. (2015). Frontal top-down signals increase coupling of auditory low-frequency oscillations to continuous speech in human listeners. *Curr. Biol.* 25 1649–1653. 10.1016/j.cub.2015.04.049 26028433 PMC4503802

[B31] Pérez A. Molinaro N. Mancini S. Barraza P. Carreiras M. (2012). Oscillatory dynamics related to the Unagreement pattern in Spanish. *Neuropsychologia* 50 2584–2597. 10.1016/j.neuropsychologia.2012.07.009 22824235

[B32] Szewczyk J. M. Schriefers H. (2013). Prediction in language comprehension beyond specific words: an ERP study on sentence comprehension in Polish. *J. Mem. Lang.* 68 297–314. 10.1016/j.jml.2012.12.002

[B33] Van Berkum J. J. A. Brown C. M. Zwitserlood P. Kooijman V. Hagoort P. (2005). Anticipating upcoming words in discourse: evidence from ERPs and reading times. *J. Exp. Psychol. Learn. Mem. Cogn.* 31 443–467. 10.1037/0278-7393.31.3.443 15910130

[B34] Wang L. Jensen O. van den Brink D. Weder N. Schoffelen J. Magyari L. (2012). Beta oscillations relate to the N400m during language comprehension. *Hum. Brain Mapp.* 33 2898–2912. 10.1002/hbm.21410 22488914 PMC6870343

[B35] Weiss S. Mueller H. M. (2012). “Too many betas do not spoil the broth”: the role of beta brain oscillations in language processing. *Front. Psychol.* 3:201 10.3389/fpsyg.2012.00201 PMC3382410 22737138

[B36] Weiss S. Mueller H. M. Schack B. King J. W. Kutas M. Rappelsberger P. (2005). Increased neuronal communication accompanying sentence comprehension. *Int. J. Psychophysiol.* 57 129–141. 10.1016/j.ijpsycho.2005.03.013 15935501

